# Multiple-Integrations of HPV16 Genome and Altered Transcription of Viral Oncogenes and Cellular Genes Are Associated with the Development of Cervical Cancer

**DOI:** 10.1371/journal.pone.0097588

**Published:** 2014-07-03

**Authors:** Xulian Lu, Qiaoai Lin, Mao Lin, Ping Duan, Lulu Ye, Jun Chen, Xiangmin Chen, Lifang Zhang, Xiangyang Xue

**Affiliations:** 1 Department of Microbiology and Immunology, Wenzhou Medical University, Wenzhou, Zhejiang, China; 2 Institute of Molecular Virology and Immunology, Wenzhou Medical University, Wenzhou, Zhejiang, China; 3 Department of Pathology, Zhuji People's Hospital of Zhejiang Province, Zhuji, Zhejiang, China; 4 Fuda Cancer Hospital Affiliated to the Medical College of Jinan University, Guangzhou, Guangdong, China; 5 Department of Obstetrics and Gynecology, Second Affiliated Hospital of Wenzhou Medical University, Wenzhou, Zhejiang, China; 6 School of Laboratory Medicine and Life Science, Wenzhou Medical University, Wenzhou, Zhejiang, China; Georgetown University, United States of America

## Abstract

The constitutive expression of the high-risk HPV E6 and E7 viral oncogenes is the major cause of cervical cancer. To comprehensively explore the composition of HPV16 early transcripts and their genomic annotation, cervical squamous epithelial tissues from 40 HPV16-infected patients were collected for analysis of papillomavirus oncogene transcripts (APOT). We observed different transcription patterns of HPV16 oncogenes in progression of cervical lesions to cervical cancer and identified one novel transcript. Multiple-integration events in the tissues of cervical carcinoma (CxCa) are significantly more often than those of low-grade squamous intraepithelial lesions (LSIL) and high-grade squamous intraepithelial lesions (HSIL). Moreover, most cellular genes within or near these integration sites are cancer-associated genes. Taken together, this study suggests that the multiple-integrations of HPV genome during persistent viral infection, which thereby alters the expression patterns of viral oncogenes and integration-related cellular genes, play a crucial role in progression of cervical lesions to cervix cancer.

## Introduction

Cervical cancer is the second leading cause of cancer-related death in women worldwide. The persistent infection by high-risk human papillomavirus (HR-HPV), such as genotype 16, 18, 31, 33, 35, 39, 45, 51, 52, 56, 58, and 59 are essential for the progression of cervical lesions [1], and over 50% cases are caused by HPV16 [2]. Viral oncoproteins, E6 and E7, of HR-HPVs contribute to cervical carcinogenesis by inactivating two major cellular tumor suppressor proteins, p53 and pRb, respectively [3]–[6]. These viral oncoproteins in infected cells can also result in chromosome instability and accumulation of mutation events [7].

A viral early promoter lied upstream of the E6 ORF, such as P97 in HPV16 [8], [9], P99 in HPV31 [10], [11] and P105 in HPV18 [12], [13], is responsible for almost all early gene expression, including E6 and E7. Upstream cis-elements in the LCR interact with cellular transcription factors and the viral transactivator/repressor E2 and regulate the transcription of HPV E6 and E7 genes [8], [14]. Furthermore, DNA methylation [15], alternative RNA splicing [9], [16], [17] and early poly(A) site polyadenylation signal [18], [19] also take part in the regulation of E6 and E7 gene expression [19].

To date, a full transcription map of oncogenic HPV16 and HPV18 in HPV-infected cells and raft tissues have been constructed [19], [20].

It's well known that the integration of HPV genomes is a key event in cervical carcinogenesis [21], [22]. Besides viral genome integration in activating cellular oncogenes or inactivating cellular tumor suppressive genes [23]–[25], HPV genome integration into host genome may change the transcription patterns of both viral and host genes [26]. It has been reported that the integration of HPV genomes can disrupt the viral E2 gene in cells and release its inhibition on the viral early promoter that controls the expression of E6 and E7 [27]. In addition, E6 and E7 transcripts cotranscribed with cellular sequences may be more stable, and thus enhance their expression level [28]–[30].

Transcription patterns of HPV16 in the tissues of cervical cancer have been reported [26], [31]. There were an episomal HPV early gene transcript (E7-E1^∧^E4) and several integrated HPV transcripts (such as E7-E1^∧^cellular RNA, E7-E1^∧^E4-cellular RNA, etc.) in HPV16-infected tissues. However, transcriptional selection in response to environmental changes is a dynamic process to achieve optimal gene expression for cell survival and carcinogenesis [32]. In this study, we applied a modified technique of amplification of papillomavirus oncogene transcripts (APOT) [26] to comprehensively explore the structure and sequences of HPV16 E7 related transcripts and their genomic annotation in 8 LSIL (low-grade squamous intraepithelial lesions), 24 HSIL (high-grade squamous intraepithelial lesions), and 8 CxCa HPV16-positive cervical biopsy samples.

## Materials and Methods

### Patients and specimens

Tissue samples of primary uterine cervical lesions containing dysplastic epithelium/tumor cells were collected from the Second Affiliated Hospital of Wenzhou Medical University (Zhejiang Province, China) from December 2010 to April 2012. The presence of HR-HPV was detected by HCII test, and the screening of HPV16 in HR-HPV-positive samples was done by HPV genotypes detection kit (KaiPu, Guangzhou, China) [33]. All of them did not receive radiation therapy or chemotherapy before operation and each patient underwent a colposcopically directed biopsy. The collected biopsy specimens were bisected. One portion was submitted for standard histopathologic diagnosis, while the other portion was stored in RNAlater (Ambion, Austin, Texas, USA) at −80°C for subsequent analysis. On the basis of the histopathologic diagnosis, the samples were divided into LSIL (CIN I, n = 8), HSIL (CIN II, n = 22; CIN III, n = 16) and cervix carcinoma (CxCa, n = 17). Additional 8 cervical tissues with normal cytology and HPV DNA negativity as controls were obtained from the patients who underwent hysterectomy owing to benign gynecologic diseases. The study has been approved by the Medical Ethics Committee of Second Affiliated Hospital of Wenzhou Medical University. All women were informed and gave their written consent to participate in the study.

### RNA and DNA Isolation from Clinical Samples

Total RNA from biopsy samples described above was isolated using TRIzol reagent (Invitrogen, Calif., USA) according to the manufacturer's instructions. To remove the residual DNA contamination, the RNA preparation was treated with Rnase-free Dnase I (Takara, Dalian, China) according to the manufacturer's protocol. Purified total RNA was dissolved in Rnase-free water and stored at −80°C. The concentration and purity of total RNA were quantified by the ultraviolet spectrophotometer at 260 nm and 280 nm and 1% agarose gel electrophoresis. Only RNA samples with an A260/A280 ratio of 1.8–2.0 and high integrity were used for the further experiment.

### Reverse Transcription and PCR Amplification of Transcripts

APOT assay reported previously was based on nested PCR reactions [26], which could only amplify the abundant transcripts and ignore the transcripts with lower levels in samples. So modified APOT assay was used to amplify the HPV oncogene transcripts. The primers for these reactions were designed according to Klaes R, et al [26]. Total RNA (1 µg) was reversely transcribed using an oligo(dT)_17_-primer coupled to a linker sequence *RT* [34] according to the manufacturer's protocol of reverse transcriptase Kit (TOYOBO, Japan). To verify first-strand cDNA quality, PCR using glyceraldehyde-3-phosphate dehydrogenase (GAPDH) -specific primers were performed as previously described [35]. First-strand cDNA encompassing viral oncogene sequences were subsequently amplified by PCR using *p1*-HPV16E7 specific primer (5′-CGGACAGAGCCCATTACAAT-3′) and linker *p0* (5′-GACTCGAGTCGACATCG-3′) as the reverse primer; and the PCR amplification was carried out in a reaction volume of 50 µl. Different from previous reports, the PCR cycles was increased to 35, and all specimens only performed one-round PCR reaction. To verify the specificity of this procedure, the “minus-RT” control in which reverse transcriptase was omitted from the reactions was also performed parallel.

### Sequence Analysis of Transcripts

The APOT amplification products were visualized by 2.5% agarose gel electrophoresis. PCR products of interest were excised from the gel and extracted using DNA agarose gel recovery kit (TianGen, Beijing, China). The corresponding amplimeres were cloned into cloning vector (TransGen, Beijing, China) and DNA sequence analysis was executed using an ABI 3730 XL Genetic Analyser (Applied Biosystems, USA) according to standard protocols. Sequencing results were analyzed using the BLASTn program provided by the National Center for Biotechnology Information, USA. Additionally, the chromosomal integration sites were ascertained using the National Center for Biotechnology Information (BLAST) and European Molecular Biology Laboratory (EBI). Moreover, the fragile sites and genes of integration sites were defined using the NCBI fragile site map viewer and the UCSC Blat tool.

## Results

### Specificity of APOT assay for HPV16 oncogene transcripts

**Figure 1:**
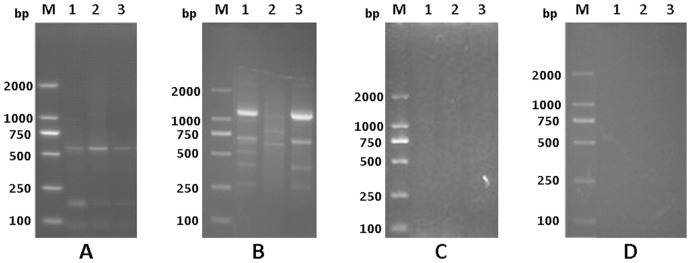
Specificity of APOT assay in detection of HPV16 oncogene transcripts. Amplified products from CaSki cells (A), HPV16-positive CxCa (B), HPV negative normal cervical tissues (C) and the “minus-RT” controls of the RNA isolated from HPV 16-positive samples (D) by the APOT assay were separated on 2.5% agarose gels. A: *Lane 1-3* were three different subcultured CaSki cells (as a positive controls); B: *Lane 1-3* were three different CxCa samples; C: *Lane 1-3* were three different normal cervical tissues (as negative controls); D: *Lane 1-3* were corresponding with samples in B, respectively; M: 250-bp DNA ladders.

The principle of the APOT assay is a 3′ rapid amplification of cDNA ends (RACE) PCR assay that achieves amplification and cloning of the region between a single short sequence in a cDNA molecule and its unknown 3′- end [34]. In general, the integrated transcripts derived from E6 and E7 oncogenes encompass viral sequences at their 5′- ends and host genome sequences at their 3′- ends [26]. The expected size of products obtained from an episome-derived transcript is 1050 bp [26] Amplimers that displayed a size different from 1050 bp may therefore be derived from an integrated HPV genome. To testify the specificity of the modified APOT assay, cDNAs from HPV16-positive Caski cell contains the integrated HPV16 genome and HPV-negative normal cervical tissues, as well as the “minus-RT” controls in which reverse transcriptase was omitted from the reactions were used. The amplified products of the cDNAs from HPV16-positive Caski cell were similar to the previous report [26], whereas no RT product was obtained from the normal cervical tissues without HPV DNA and the “minus-RT” control (Figure 1). These data indicated the modified APOT assay can specifically detect the transcripts derived from the integrated HPV genome.

### Characteristics of HPV16 oncogene transcripts in the tissues of cervical intraepithelial neoplasia and cervix carcinoma

To analyze the HPV16 oncogene transcripts, 40 HPV16-positive cervical specimens (LSIL, n = 8; HSIL, n = 24; CxCa, n = 8) with good quality RNA were selected among 63 collected samples in this study. Total 133 transcripts containing viral fragments were found. Among these transcripts, 64 fragments had HPV16 E7-E1* sequences at their 5′- ends and directly connected with poly A at their 3′- ends (Figure S1). Furthermore, there were four different disruptions of E1 region at nt 880, 949, 1054 and 1234 (Figure S2). The transcripts containing an E1-splice donor signal at nt 880 [36] might belong to potential episomal pattern, whereas the transcript which truncated at nt949 might be a result of internal priming by oligo dT [37]. Other transcripts which truncated at nt 1054 and 1234 neither contained poly A sequences, nor any polyadenylation site belong to viral or host, so these transcripts were viewed as potential integrated patterns. In addition, we also found another transcript which has E7 ORF spliced at nt 880 to the E4-splice acceptor site at nt 3358 and then spliced from the E4-splice donor signal at nt 3632 to the L1-splice acceptor site at 5639, and also terminated at poly A (Figure S1). In this transcript, the E4 ORF is not disrupted. Lack of a splice donor signal at nt 5815 in this transcript indicates that the HPV16 genome disrupted at nt 5815 might also take part in the virus genome integration.

**Figure 2:**
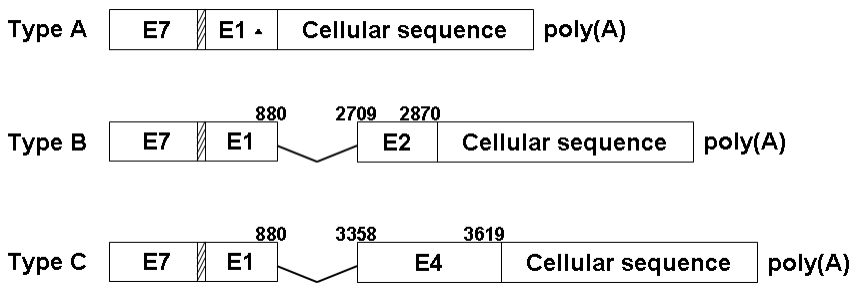
Three types of HPV 16 early gene transcription pattern. Type A shows E1 sequences spliced directly to cellular flanking sequence; Type B shows E1 spliced to E2, with E2 fused with a cellular sequence; Type C shows E1 spliced to E4, with E4 running into a cellular sequence.^ ▴^, there are two integration sites in E1 (data shown in Figure S3). The boxes within slashes represent six nucleotides between E7 and E1gene.

In addition, there were 64 viral transcripts directly connected to host genome sequences and they were all began with the beginning of the forward primer (p1) at nt 729. These HPV16 oncogene integrated transcripts could be divided three different types (Figure 2). Among these transcripts, Type A has HPV16 E7-E1^*^ sequences at their 5′- ends and directly connected to host genome sequences. However, there were two different integration sites of E1 region (at nt880 and nt1107) in this type (Figure S3). The site at nt880 contained an E1-splice donor signal while the site truncated at nt1107 might be more likely to linearize the viral circular genome for integration into the host genome. Transcript type B has an E2 ORF disrupted at nt2870 and the Type B sequence composes of HPV16 E7-E1^∧^E2^*^ at its 5′- ends and the host genome sequence at its 3′- ends. In transcript type C, the E1^∧^E4 stop codon is disrupted for virus integration and an entire E1^∧^E4 ORF without a stop codon is fused in frame to host sequence. Among these three patterns, transcripts of Type A and C had been reported by Wentzensen N, et al. [31]. However, transcript of Type B had not previously been reported in precancerous lesions and cervical cancer.

**Figure 3:**
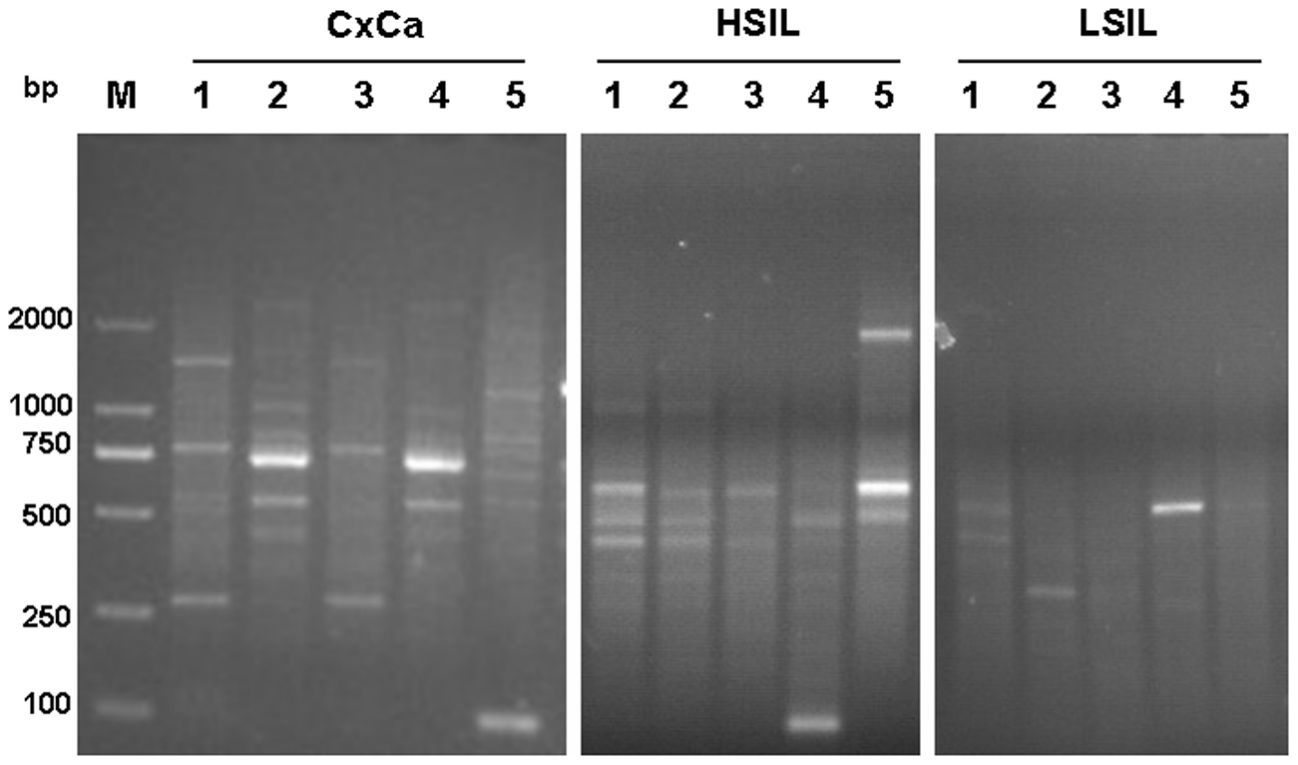
Detection of HPV 16 early gene transcripts by the APOT assay. HPV16-positive clinical samples with LSIL, HSIL and CxCa were subjected to the APOT assay and separated on 2.5% agarose gels, *Lane 1-5* mean five different samples in each pathological type. M: 250-bp DNA Ladders.

**Figure 4:**
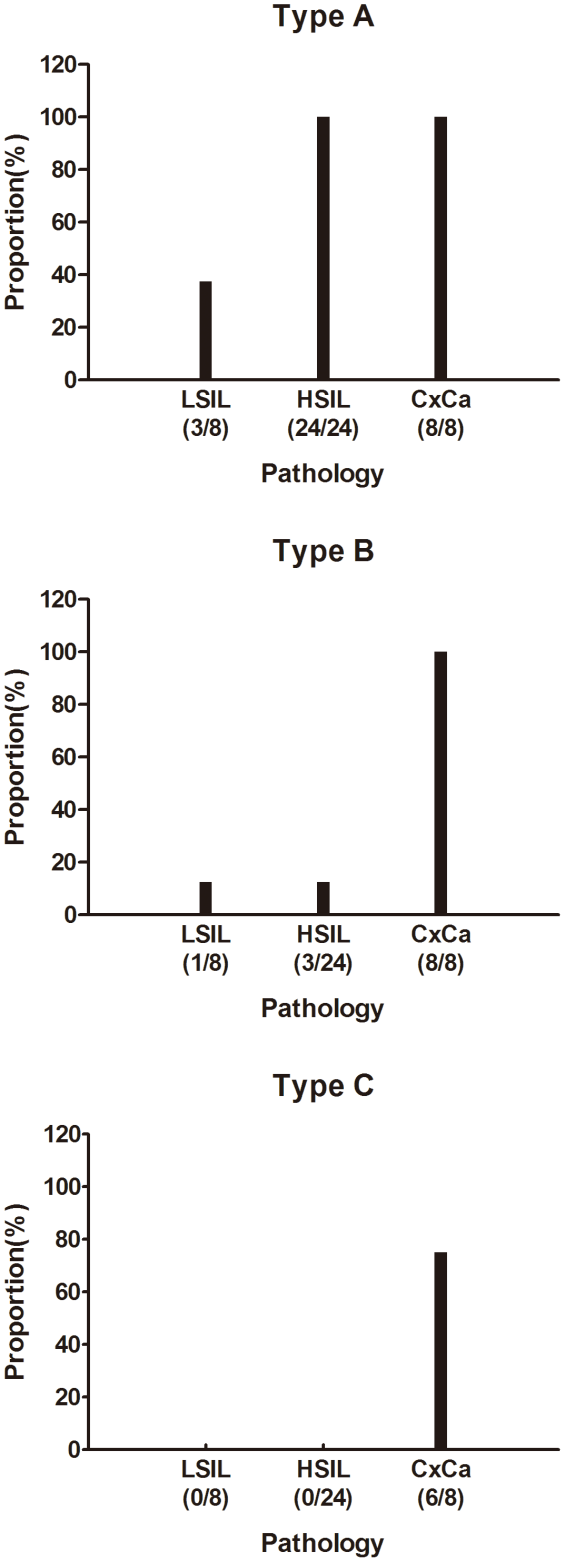
The proportion of patient samples that contain the different type of transcripts.

Moreover, HPV16 oncogenes showed significantly different transcription patterns in the tissues of LSIL, HSIL and CxCa (Figure 3, 4 and Table 1). Among these 3 transcription patterns detected in our patients, the Type A and Type B were higher prevalence than Type C, which were observed in almost all pathological types, whereas the Type C was detected only in the samples of CxCa, with a detection frequency of 75% (Table 1 and Figure 4). All patient samples displayed the Type A, but all CxCa samples had the Type B and Type C (Figure 4). Consistent with the presumption of potential integration of the viral genome in the later stages of cancer development [38], [39], the prevalence of fusion transcripts were higher in HSIL and CxCa than LSIL.

**Table 1:** The Transcript Number of Three Transcription Patterns in the Groups of LSIL, HSIL and CxCa.

			Different pathological types	
Pattern	Total	LSIL	HSIL	CxCa
Type A	48	3	35	10
Type B	10	1	1	8
Type C	6	-	-	6

64 transcripts in total, directly connected to host genome sequences at their 3′- ends, were detected from 40 HPV 16-positive cervical specimens, including 8 LSIL, 24 HSIL and 8 CxCa. Type A and B were detected in all HPV 16-positive samples while type C was only found in CxCa. “-”, no transcripts.

### Integration sites and characterization of the cellular flanking sequence

To identify the individual chromosomal locations, all 64 fusion-transcripts containing viral and cellular sequences were further analyzed by BLASTn comparisons to the whole genome database. Our data show that all chromosomes, except for Chr21 and X, were integrated with HPV16 genome, confirming the previous reports that no preferential HPV integration site was seen in selection of the human chromosome [40]. Some loci, such as 1p36.22, 1p36, 2p24, 2q33, 5q31.1, 5q31, 6p24, 8p23, 10q22.1, 13q22.1, 19q13 and 19p13.3, were reported previously [31], [41]–[45] (Table 2). Among these integration events, fourteen of 40 samples exhibited multiple integration sites (Table 2). Although local DNA rearrangements could happen frequently and rapidly after the integration [43], we found that cellular flanking sequences in 11 tissues were mapped to different chromosomes, indicating the presence of multiple independent integrations in these samples. Moreover, we found that multiple-integration events were significantly higher in CxCa tissues (75%) than in the cervical tissues of LSIL (50%) and HSIL (53.8%). Screening of all integration loci indicates that 35 of 63 mapped integration sites were located in or close to a fragile site with a distance of 26 bp to 5 Mbp (Table 2). Among the 22 mapped fragile sites, FRA13A was found in 4 independent samples. Twenty-two transcripts were not associated with any fragile site.

**Table 2:** Summary of All Integration Sites and Characterization of the Cellular Flanking Sequence.

Sample ID	Pathology	Integration locus	Nearest fragile site*	Accession code*	Gene name	Nearest genes*	Integrate to
79	LSIL	11q13	-	PRDX5	peroxiredoxin 5	ESRRA  CCDC88B	n.a
		11q23.3	-	AMICA1	adhesion molecule, interacts with CXADR antigen 1	MPZL3  SCN2B	Exon 9
196	LSIL	11q23.3	-	AMICA1	adhesion molecule, interacts with CXADR antigen 1	MPZL3  SCN2B	Exon 9
184	HSIL	10q22.1	FRA10D(10q22.1)	KIAA1279	KIAA1279	DDX21  ACTBP14	Intron 7
179	HSIL	8p23	-	ARHGEF10	Rho guanine nucleotide exchange factor (GEF) 10	MIR596  KBTBD11	Intron 27
		8p23.3	-	*LOC100131395*	LOC100131395	-	n.a.
		8q23.1	-	*ANGPT1*	angiopoietin 1	PGAM1P13  HMGB1P46	Intron 5
		8q23	-	*EBAG9*	estrogen receptor binding site associated, antigen, 9	PKHD1L1  SYBU	n.a.
120	HSIL	19q13.1	FRA19A (19q13)	COX6B1	cytochrome c oxidase subunit VIb polypeptide 1	ETV2  UPK1A	n.a.
		19q13.13	FRA19A (19q13)	*UPK1A*	uroplakin 1A	COX6B1  ZBTB32	n.a.
		12q23.3	FRA12E (12q24)	SLC41A2	solute carrier family 41, member 2	KRT18P20  CHST11	Exon 10
55	HSIL	1p36.22	FRA1A (1p36)	*MIR34A*	microRNA 34a	LOC727721  GPR157	Intron 2
169	HSIL	15q21.3	-	RFX7	regulatory factor X, 7	HMGB1P33  CD24P2	n.a.
29	HSIL	13q14.11	FRA13A(13q14)	MRPS31	mitochondrial ribosomal protein S31	SLC25A15  FOXO1	Exon 1
		13q14.3	FRA13A(13q14)	THSD1	thrombospondin, type I, domain containing 1	VPS36  TPTE2P2	n.a.
		17q25.1	-	EXOC7	exocyst complex component 7	FOXJ1  ZACN	Intron 6
203	HSIL	2p24	FRA2C(2p24.2)	*ROCK2*	Rho-associated, coiled-coil containing protein kinase 2	LOC650157  PQLC3	Intron 1
		2p21	FRA2C(2p21)	*MSH2*	mutS homolog 2	EPCAM  LOC644093	n.a.
139	HSIL	16q22.1	FRA16C(16q22.1)	COG4	component of oligomeric golgi complex 4	SF3B3  FUK	Intron 10
		16q23.3	FRA16D(16q23.2)	*WWOX*	WW domain containing oxidoreductase	LOC100131126  MAF	Intron 9
		16q23	FRA16D(16q23.2)	ADAMTS18	ADAM metallopeptidase with thrombospondin type 1 motif, 18	NUDT7  VN2R10P	Intron 4
		16q24.2	FRA16D(16q24)	MAP1LC3B	microtubule-associated protein 1 light chain 3 beta	FBXO31  NR3C1P1	Intron 3
		7q36	-	LMBR1	limb development membrane protein 1	NOM1  RNF32	Intron 5
		7p14.3	FRA7B(7p14)	*NT5C3A*	5′-nucleotidase, cytosolic IIIA	RP9  FKBP9	Intron 7
		9q33	FRA9E(9q32)	*TNC*	tenascin C	DEC1  TNFSF8	Intron 3
		9q21.33	-	*DAPK1*	death-associated protein kinase 1	C9orf170  CTSL	Intron 3
68	HSIL	10pter-q25.3	FRA10D(10q25)	ASCC1	activating signal cointegrator 1 complex subunit 1	ANAPC16  SPOCK2	Intron 6
		10q21.2	-	ZNF365	zinc finger protein 365	RTKN2  ADO	Intron 5
		18q21.1	FRA18B(18q21.3)	SETBP1	SET binding protein 1	KRT8P5  LOC10013166	Intron 3
155	HSIL	6p24	-	*MAK*	male germ cell-associated kinase	GCM2  LOC100506379	Intron 8
C15	HSIL	19q13	FRA19A(19q13)	ACTN4	actinin, alpha 4	EIF3K  CAPN12	Intron 1
51	HSIL	4q32-q33	FRA4C(4q33)	TLL1	tolloid-like 1	LOC646995  SPOCK3	Intron 2
		5q31	FRA5C(5q31.1)	PCDHA@	protocadherin alpha cluster, complex locus	LOC100421074  PCDHB1	Intron 1
		5q31.1	FRA5C(5q31.1)	*Egr1*	early growth response 1	Reep2  RPL7P19	Intron 1
28	HSIL	14q21.2	FRA14A(14q21)	*FANCM*	Fanconi anemia, complementation group M	SNORD127  MIS18BP1	Intron 4
42	CxCa	11q13	-	PRDX5	peroxiredoxin 5	ESRRA  CCDC88B	n.a.
		13q22.1	FRA13C(13q21.2)	PIBF1	progesterone immunomodulatory binding factor 1	BORA  PSMD10P3	n.a.
		22q12.3	FRA22B (22q12.2)	*TIMP3*	TIMP metallopeptidase inhibitor 3	FBXO7  LARGE-AS1	Intron 2
97	CxCa	11q13	-	PRDX5	peroxiredoxin 5	ESRRA  CCDC88B	n.a.
		2q37	FRA2J(2q37.3)	AGAP1	ArfGAP with GTPase domain, ankyrin repeat and PH domain 1	Loc642692  Gbx2	Intron 14
		13q14.11	FRA13A(13q14)	MRPS31	mitochondrial ribosomal protein S31	SLC25A15  FOXO1	Exon 1
6	CxCa	2q33	FRA2I(2q33)	*CD28*	CD28 molecule	LOC100287498  KRT18P39	n.a.
		2p22-p21	FRA2C(2p21)	EIF2AK2	eukaryotic translation initiation factor 2-alpha kinase 2	SULT6B1  HEATR5B	Intron 2
		1q25.3	FRA1G(1q25)	CACNA1E	calcium channel, voltage-dependent, R type, alpha 1E subunit	GM140  ZNF648	n.a.
		11q13	-	PRDX5	peroxiredoxin 5	ESRRA  CCDC88B	n.a.
206	CxCa	3p14.3	FRA3B(3p14.2)	*DENND6A*	DENN/MADD domain containing 6A	PDHA1P1  ARF4	n.a.
		7q21.11	FRA7E(7q21.2)	SEMA3E	sema domain, immunoglobulin domain (Ig), short basic domain, secreted, (semaphorin) 3E	SEMA3A  PCLO	Intron 2
94	CxCa	11q13	-	PRDX5	peroxiredoxin 5	ESRRA  CCDC88B	n.a.
213	CxCa	8q23	-	*EBAG9*	estrogen receptor binding site associated, antigen, 9	PKHD1L1  SYBU	n.a.
		4p13	-	RHOH	ras homolog family member H	N4BP2  CHRNA9	Intron 9
131	CxCa	13q22.1	FRA13C(13q21.2)	PIBF1	progesterone immunomodulatory binding factor 1	BORA  PSMD10P3	Exon 18
		13q14.11	FRA13A(13q14)	MRPS31	mitochondrial ribosomal protein S31	SLC25A15  FOXO1	Exon 1
		19p13.3-p13.2	FRA19B(19p13)	INSR	insulin receptor	LOC100996504  MBD3L3	Intron 3
		19q12	-	*CCNE1*	cyclin E1	PLEKHF1  LOC126170	n.a.
		11q13	-	PRDX5	peroxiredoxin 5	ESRRA  CCDC88B	n.a.
190	CxCa	13q14.11	FRA13A(13q14)	MRPS31	mitochondrial ribosomal protein S31	SLC25A15  FOXO1	Exon 1
		9q21.33	-	*DAPK1*	death-associated protein kinase 1	C9orf170  CTSL	Intron 3

*“-”, no entry of fragile sites or nearest genes; n.a., not applicable because fusion transcript is in antisense orientation.

* Genes highly relevant to cervix cancer which located in integration sites are indicated in italics, and genes indicated by an underline shows they are related to tumor.

The cellular flanking sequences of viral-cellular fusion transcripts were further examined for known genes. Most of these fused transcripts had a cellular sequence from the coding orientation of known genes and thirty transcripts had the cellular sequence from an intron region, and 8 transcripts were fused with a sense exon sequence of the predicted genes (Table 2). Among these predicted genes, *AMICA1*, *DAPK1*, *EBAG9*, *PIBF1* were affected twice, *MRPS31* four times and *PRDX5* even six times by the viral integration. At the same time, the nearest host genes to each integration site in the direction of transcription were also analyzed (Table 2). Among these predicted genes integrated or closed to the integration site, we identified several tumor-associated genes, including *PRDX5*, *CD28*, *ROCK2*, *RHOH*, *TIMP3* and *DAPK1*, etc. As shown in Table 1, the transcripts type D and E were only detected in CxCa and most of their integration loci were located in or close to the fragile sites of FRA13C, FRA22B, FRA2I and FRA13A. The genes associated with the transcripts type D and E were oncogenes (*CD28* and *EBAG9*), tumor suppressor genes (*TIMP3*), or tumor-related genes (*PIBF1*and *MRPS31*).

## Discussion

Integration of HPV genome into host chromosomes represents an early clonal event to provide an additional selective advantage for the expansion of the neoplasm. Viral transcripts have been detected by the APOT assay [26], [31], [42]–[45]. Although APOT assay has some advantages in detection transcripts from each chromosome integration site, there are several limitations. First, it is difficult to amplify very long integration-derived transcripts, which will underestimate the number of tumors with integrated HPV DNA [45]. Second, APOT is one type of nested PCR, which may tend to amplify the transcripts with higher levels and ignore those with lower levels. Third, It has been reported that the internal poly A priming could replace the oligo(dT) primer within certain limits, and generating a set of anchored oligo(dT) primers for cDNA synthesis. These sequences caused by internal priming interrupted the generating of full-length cDNA and confused the analysis of alternative splicing [37]. With our modified APOT assay to detect the transcription pattern of the cervical tissues, we did find many viral transcripts connected with poly A or host genome sequences in HPV16-infected cervical squamous epithelial tissues. We noticed that there were a lot of viral transcripts directly ended with poly A at their 3′- ends. Except for the reported E1-splice donor signal site (nt 880), the truncation sites at nt1054, 1234 and 5815 neither contained internal poly A sequences nor any polyadenylation signals should be potential novel integrated sites and need for further analysis. The viral-cellular fusion transcript of type A and C has been reported previously [26], [31], [41]. In the Type C transcript, the integration disruption of E4 termination codon would result in the E4 to use a host termination codon. In this study, we also noticed that some cervical cancer samples contained all three types of transcripts were viral-cellular fusion transcripts.

HPV16 transcription patterns in LSIL, HSIL, and CxCa were significantly different. We found that the Type C transcript was only detected in the samples of CxCa and more random integration sites existed in our tissue samples. Similar to previous reports [31], [38]–[42], [46], [47], our study indicates that HPV integration has no preferential site in the human genome. Except for chromosome 21 and X, other chromosomes are all susceptible to HPV16 integration. Approximately 55% integrations are located in or close to a fragile site. Different from previous reports [42], [45], we noticed that integration events often occur multiple times significantly more in cervical cancer than in LSIL and HSIL. These data not only provide biological support to the epidemiologic observation that persistent infection by specific types of HR-HPV is the important cause of cervical carcinoma [1], but also indicate that subsequent selection for and accumulation of mutations in yet-to-be-identified key cellular regulatory genes promotes further progression to cervical cancer.

The integration not only changes the transcription pattern relevant for the dysregulated expression of the viral oncogenes, but also affects the expression of the host gene with virus genome integration. The integration alters the expression of host genes in integration sites, even if this occurs within the intron sequences [43], [45]. In our study, we identified a broad spectrum of cancer-associated genes in the integration sites and flanking sequence regions. Most of genes in the integration sites were associated with tumor development, and nineteen genes were strongly related to cervical cancer. Some of them act as tumor suppressors (such as, *miR-34a*, *MSH2*, *WWOX* and *TIMP3*, *et al*) or oncogenes (such as, *ROCK2*, *CD28*, *EBAG9* and *ANGPT1*, *et al*). Interestingly, most of them were not reported in previous documents [31], [45]. *MiR-34a*, an important tumor suppressor, is down-regulated in cervical cancer [48], [49]. It has been reported that oncoprotein E6 of HPV16 and HPV18 can inhibit the expression of tumor-suppressive *miR-34a* by destabilization of p53 and resulted in cell proliferation [50]. The disruption of *miR-34a* gene might further interpret the phenomenon of reduced expression of *miR-34a* in cervical cancer. *MSH2* is a DNA mismatch repair protein, and associated with DNA repair pathway [51], [52]. Decreased expression of MSH2 might be a risk factor in the early stage cervical cancer [53]. *ROCK2*, an important signaling molecule, can promote cervical cancer metastasis by upregulating and activating the expression and function of moesin protein through RhoA/ROCK2 pathway [54]. Besides the cancer-associated genes, the genes in integration sites and flanking sequence regions might be also beneficial for viral genome integration. *FANCM* which is a DNA translocase and highly related to DNA replication regulates checkpoint signaling and replication fork progression [55], [56]. Other genes, such as *COX6B1* is related to cell apoptosis [57] and *ESRRA* also have been reported associated with cervical cancer [58]. In addition, among 45 integration events, 13 events led to antisense transcription of the coding sequences, such as *PRDX5*, *EBAG9* and *CD28, etc*. These integrations were generally deemed of no interest. However, their sense sequences were associated with DNA restoration or tumor development and might affect both host and viral gene expression during the development of cervical cancer. The most integration in the antisense orientation was the gene encoding peroxiredoxin 5 (*PRDX5*), a protective emzyme against oxidative stress [59], [60]. Its altered expression due to HPV16 integration could have significant virological consequence, along with the integration into DNA repair genes, such as FANCM and MSH2. Upregulation of *EBAG9* expression has been observed in several malignant tumors [61]. The synergistic stimulation factor of *CD28* which maintains immune homeostasis plays a role in increasing susceptibility to cervical cancer [47].

In conclusion, changes of the transcription patterns of HPV 16 early genes go along with the progression from cervical intraepithelial neoplasia to cervix carcinoma and viral genome integration into host chromosome. The change or selection of transcription patterns and the integration on the expression of host genes in the integration sites and flanking cellular sequence regions might all take part in oncogenesis of HPV16-induced cancers.

## Supporting Information

Figure S1 **The types of viral sequences connected with poly A at their 3′- ends.** The type of Class I shows E1 sequences directly ended with poly A; the type of Class II shows E1 spliced to E4 and then to L1 and also ended with poly A sequences. ^▴^, there are several truncation sites in E1 (data shown in Figure S2).
(TIF)

Figure S2 **Different truncation sites in E1 region in the type of Class I.** There are four truncated sites in E1, 880, 949, 1054 and 1234, respectively.
(TIF)

Figure S3 **Several spliced donor sites in E1.** In Type A there are two integration sites in E1, 880, and 1107, respectively. The solid boxes mean cellular sequences.
(TIF)
