# Supplementary figures and images for: Multiple-Integrations of HPV16 Genome and Altered Transcription of Viral Oncogenes and Cellular Genes Are Associated with the Development of Cervical Cancer
Source: PLoS One. 2014 Jul 3;9(7):e97588. doi: 10.1371/journal.pone.0097588 (PMC4081011; doi:10.1371/journal.pone.0097588)

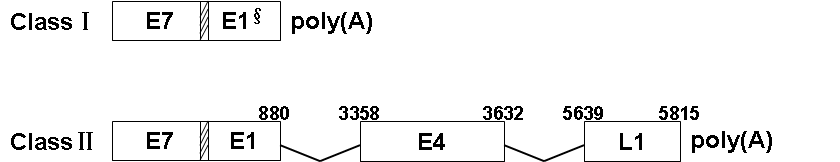

Supplement: Figure S1 — The types of viral sequences connected with poly A at their 3′- ends. The type of Class I shows E1 sequences directly ended with poly A; the type of Class II shows E1 spliced to E4 and then to L1 and also ended with poly A sequences. ▴, there are several truncation sites in E1 (data shown in Figure S2). (TIF) [file pone.0097588.s001.tif]

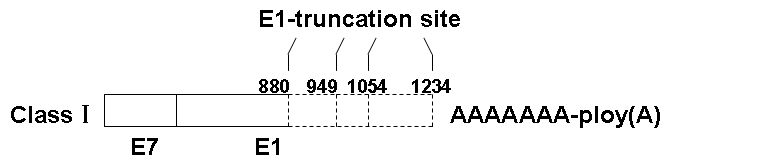

Supplement: Figure S2 — Different truncation sites in E1 region in the type of Class I. There are four truncated sites in E1, 880, 949, 1054 and 1234, respectively. (TIF) [file pone.0097588.s002.tif]

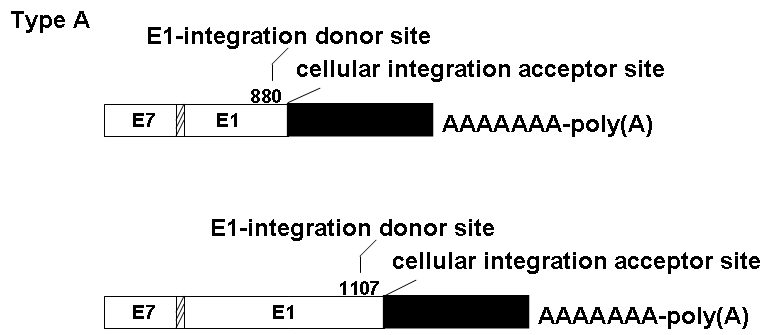

Supplement: Figure S3 — Several spliced donor sites in E1. In Type A there are two integration sites in E1, 880, and 1107, respectively. The solid boxes mean cellular sequences. (TIF) [file pone.0097588.s003.tif]
